# Endometrial stromal tumors: immunohistochemical and molecular analysis of potential targets of tyrosine kinase inhibitors

**DOI:** 10.1186/2045-3329-3-3

**Published:** 2013-03-07

**Authors:** Ruth Sardinha, Teresa Hernández, Susana Fraile, Francesc Tresserra, August Vidal, Maria Carmén Gómez, Aurora Astudillo, Nieves Hernández, Javier Saenz de Santamaría, Jaume Ordi, Luis Gonçalves, Rafael Ramos, Carmen Balañá, Enrique de Álava

**Affiliations:** 1 Pathology Department, Hospital Espírito Santo E.P.E, Évora, Portugal; 2 Centro de Investigación del Cáncer-IBMCC USAL-CSIC, Salamanca, Spain; 3 Pathology Department, USP-Institut Universitari Dexeus, Barcelona, Spain; 4 Pathology Department, Hospital de Bellvitge, Barcelona, Spain; 5 Pathology Department, Hospital Universitari Germans Trias i Pujol, Badalona, Spain; 6 Pathology Department, Hospital Universitario Central de Asturias, Oviedo, Spain; 7 Anatomical Pathology Department, Hospital Universitario de la Laguna, Canarias, Spain; 8 Pathology Department, Complejo Hospitalario Universitario de Badajoz, Badajoz, Spain; 9 Pathology Department, Hospital Clinic de Barcelona, Barcelona, Spain; 10 Pathology Department, Hospital do Espírito Santo E.P.E, Évora, Portugal; 11 Pathology Department, Hospital Son Espases, Palma de Mallorca, Spain; 12 Medical Oncology Service, Catalan Institute of Oncology - Hospital Germans Trias i Pujol, Badalona, Spain

**Keywords:** Endometrial stromal tumors, Tyrosine kinase inhibitors, KIT, PDGFRA, EGFR, Systemic treatment

## Abstract

**Background:**

The systemic treatment of malignant endometrial stromal tumors (EST) is not well established. A few reports describe objective responses to imatinib, which suggest a novel therapeutic strategy for these tumors. Due to these facts, we aimed to perform a retrospective analysis of possible molecular targets of tyrosine kinase inhibitors (TKI) in EST: KIT, PDGFRA and EGFR.

**Methods:**

52 endometrial stromal sarcomas and 13 undifferentiated endometrial sarcomas were examined and reviewed. Mutational analysis were performed for exons 9, 11, 13, and 17 of the *KIT* gene, exons 12 and 18 of the *PDGFRA* gene and exons 18, 19, 20 and 21 of the *EGFR* gene. The incidence and distribution of the KIT, PDGFRA, and EGFR expression were examined by immunohistochemistry, and *EGFR* amplification was assessed by fluorescence *in situ* hybridization.

**Results:**

No mutations in *KIT*, *PDGFRA* and *EGFR* genes were detected. Overexpression of KIT, PDGFRA, EGFR, was detected in 2 (3%), 23 (35.4%), 7 (10.8%) cases respectively, whereas amplification of *EGFR* gene was not found.

**Conclusions:**

Absence of significant expression, amplification and activating mutations on these tyrosine kinase receptors suggest that it is unlikely that EST can benefit from therapies such as TKI on the systemic setting.

## Background

Endometrial stromal sarcoma, low grade (ESS) and undifferentiated endometrial sarcoma (UES) belong to the rare group of endometrial stromal tumors (EST), which represents 15% of uterine sarcomas [1]. ESS presents a cellular background similar to the cells of normal endometrial stroma in proliferative phase. In contrast, UES lacks specific differentiation and bears no histological resemblance to endometrial stroma. Tumor cells are high-grade spindle to polygonal-shaped, with marked nuclear pleomorphism and high mitotic activity. Necrosis and vascular invasion are commonly seen [2]. While ESS is characterized by indolent course and late recurrences, with a 5-year overall survival (OS) up 70%, UES is usually diagnosed at advanced stages, and has a high rate of distant metastasis and a 5-year OS ranging from 25-55% [3-6]. FIGO stage [7] is the strongest prognostic factor for these malignancies [5,8]. CD10 is the most sensitive marker for ESS [9,10]. Estrogen and progesterone receptors [11] and aromatase [12] are usually expressed in ESS, and less commonly in UES [13-15]. The rearrangement *t*(7;17)(p15;q21), which results in *JAZF1/JJAZ1* gene fusion, is the cytogenetic hallmark of ESS [16], although other translocations have been reported [17-19]. In contrast, UES is characterized by a complex karyotype [20,21]. Recently, the *t*(10;17)(q22;p13) that results in *YWHAE-FAM22A/B* gene fusion with oncogenic properties was reported in a subset of UES [22], which is associated to the expression of Cyclin D1 [23,24] and β-catenin [23]; this supports the recent sub-classification of UES [15,25]. Surgery is the standard treatment, and includes total hysterectomy and bilateral salpingo-oophorectomy. However, due the rarity of these tumors, distinct clinical behavior, and lack of randomized studies including both categories, an appropriate systemic treatment of these malignancies was not been yet established.

Molecular targets of tyrosine kinase inhibitors (TKI) such as imatinib mesylate (Glivec^®^, STI-571, Novartis, Switzerland), gefitinib (Iressa^®^, AstraZeneca, Macclesfield, UK) and erlotinib (Tarceva^®^, OSI-Pharmaceuticals, New York, NY), which includes PDGFRA/B, KIT, C-ABL and EGFR, were reported to be expressed in ESS and UES by immunohistochemistry [26-40], although without presence of any activating mutations [36-39,41,42]. Interestingly, a few reports described objective responses with imatinib in patients who express at least one TKI target [36,37,43]. Another report described a unique case of UES with EGFR expression and *EGFR* amplification which temporarily responded to imatinib [42]. Based on these findings, an extensive evaluation of the molecular targets of TKI on EST was carried out to identify a novel therapeutic strategy for these malignancies. In the present study we analyzed the gene status and protein expression of KIT, PDGFRA, and EGFR in a large series of ESS and UES to evaluate their distribution among the distinct subgroups and correlate the immunohistochemical expression with mutational status.

## Material and methods

### Patient selection and study design

A series of 75 EST was retrieved from Spanish centers associated to Spanish Sarcoma Group (GEIS) and from the Pathology Departments of Complejo Hospitalario Universitario de Badajoz, Badajoz, Spain and Hospital do Espírito Santo E.P.E, Évora, Portugal, and sent to Tumor Bank of the Cancer Research Centre – Salamanca, Spain. The selection of patients was made according to the following inclusion criteria - previous diagnosis of EST (any histological grade) and availability of histological material sufficient to perform the study. After receiving and encoding the samples, cases were reviewed and subclassified by 1 co-author (EA) based on the current WHO classification [2]. The study was approved by the Ethics Committee of Hospital Germans Trias i Pujol (Spain), and was conducted in accordance with the Declaration of Helsinki and Spanish regulative law for Tumor Banks.

### Tissue microarray and immunohistochemistry

65 samples were considered valid and included in the study. Ten cases were discarded because of a sample too small to perform the proposed study or non-representative sample or diagnosis different than EST. Before tissue microarray (TMA) construction, representative areas of tumor were selected on hematoxylin and eosin (H&E) section and marked on the paraffin block. For each sample were obtained two cylinders of 1 mm diameter and placed in a recipient block using a tissue microarrayer (Manual Tissue Array; Beecher Instruments Inc. Sun Prairie, Wisconsin, USA). In total two TMAs were constructed according to previously described [44].

The tyrosine kinase receptors (TKR) evaluated were KIT, PDGFRA and EGFR, and to confirm diagnosis the expression of two markers most commonly used on ESS [45,46], CD10 and Calponin were assessed in each tumor. Immunohistochemistry (IHC) was performed in 3 μm sections. KIT, PDGFRA, CD10 and Calponin immunostaining was performed using a Discovery^®^ Ventana automated immunostainer (Ventana Medical Systems, Tucson, Arizona, USA). Heat-induced antigen retrieval was done with Tris-EDTA buffer (pH 8.0) for KIT, PDGFRA and Calponin and with citrate buffer (pH 6.0) for CD10. Sections were incubated with the primary antibodies Calponin (clone CALP; Dako, Carpinteria, CA, USA), KIT (polyclonal; Dako, Carpinteria, CA, USA), PDGFRA (polyclonal; Thermo Fisher Scientific, Fremont, CA, USA) and CD10 (clone 56C6; Novocastra Laboratoires, Newcastle upon Tyne, UK) at 1:100 dilution. Universal secondary biotinylated antibody (Discovery™ Universal Secondary Antibody, Ventana Medical Systems, Tucson, Arizona, USA) was used and developed for detection using the DAB MAP system (Ventana Medical Systems, Tucson, Arizona, USA). For EGFR analysis expression, a *ready-to-use* monoclonal antibody (clone 2.1E1; Gennova Scientific, Seville, Spain) was used. Briefly, paraffin-embedded sections were deparaffinized in xylene and rehydrated in downgraded alcohols and distilled water. Antigen retrieval was performed with Proteinase K solution (Dako, Carpinteria, CA, USA). The primary antibody was detected using a secondary antibody- horseradish peroxidase polymer conjugate (Dako REAL™ EnVision™ Detection System; Dako, Carpinteria, CA, USA), and all incubations were done with the Dako Autostainer Plus system (Dako, Carpinteria, CA, USA). All sections were counterstained with hematoxylin, upgraded alcohols, and xylene, mounted, and analyzed by standard light microscopy.

### Immunohistochemical evaluation

The expression of CD10 and Calponin was considered positive when over 1% of the tumor cells showed cytoplasmic expression. The TKR expression was detected in the cytoplasm of tumor cells, and each case was interpreted for immunoreactivity using a 0 to 3 semiquantitative scoring system for both the intensity of stain and the percentage of positive cells, as previously reported [47]. The multiplicative index of intensity and labeling was considered for statistic analysis and the expression was defined as weak and/or focal for a multiplicative index 1–3, moderate local or diffuse for a multiplicative index 4–6 and intense and diffuse if the multiplicative index was >6. The IHC analysis was scored by the same co-author who performed the histologic review (EA).

### Molecular analysis of tyrosine kinase receptors

#### DNA extraction

Ten serial 5 μm sections were cut and transferred into 15 ml conical centrifugation tubes and deparaffinized in xylene (two times for 5 minutes) and absolute ethanol (two times for 5 minutes) at 4000 rpm, followed by drying of the samples at room temperature. Subsequently, DNA extractions were performed according to QIAamp DNA Mini Kit protocol (Qiagen, Hilden, Germany), and then quantified by spectrophotometry (SmartSpec Plus Spectrophotometer, Bio-Rad Laboratories Inc., CA, USA).

#### *KIT* and *PDGFRA*

According to previous studies [48-50], exons 9, 11, 13, and 17 of *KIT* gene and exons 12 and 18 of *PDGFRA* gene were amplified in order to identify possible mutations. Amplification of *GAPDH* gene was performed to confirm integrity and quality of extracted DNA. PCR amplification of *KIT* and *PDGFRA* was performed using 1–5 μl of genomic DNA, 1X PCR buffer + 2 mM MgCl2 (Roche Applied Science, Mannheim, Germany), 0.15 mM of each primer, 200 μM of each dNTP (GeneAmp^®^ dNTP Blend, 10 mM, ABI, Carlsbad, CA, USA), and 1.5 U of Taq DNA polymerase (Roche Applied Science, Mannheim, Germany) in a total volume of 25 μl. The PCR conditions were 94°C for 10 min, 40 cycles of 1 min at 94°C, 1min30sec to 56°C (*KIT*) or 65°C (*PDGFRA*) and 1 min at 72°C, followed by a cycle of 10 min at 72°C. The amplified products were visualized on agarose gel 2%.

Prior to sequence analysis, *KIT* and *PDGFRA* PCR products were first purified using a QIAquick^®^ PCR Purification Technology Kit (Qiagen, Hilden, Germany). Direct sequencing of PCR products was performed using ABI PRISM^®^ BigDye Terminator Cycle Sequencing Kit in an ABI Prism 3100 Genetic Analyzer (Applied Biosystems, Carlsbad, CA, USA) according to the manufacturer instructions. Sequencing primers were the same as those used for PCR, and both strands (forward and reverse) were sequenced.

All sequences obtained were visualized and analyzed in the program Sequence Scanner Software v1.0 (Applied Biosystems, Carlsbad, CA, USA), based on the reference sequences: *KIT* - ENSG00000157404 and *PDGFRA* - ENSG00000134853.

#### EGFR

PCR amplification of exons 18, 19, 20 and 21 of the *EGFR* gene, which encompass most of the *EGFR* mutations [51,52], was carried out using previously described primers [53,54] (Table 1). Using 4 μl of DNA, PCR was performed in a reaction volume of 15 μl containing 1X PCR buffer (Applied Biosystems, Carlsbad, CA, USA), 2.5 mM MgCl2 (Applied Biosystems, Carlsbad, CA, USA), 0.17 mM of each primer, 200 mM of each dNTP (GeneAmp^®^ dNTP Blend, 10 mM, Applied Biosystems, Carlsbad, CA, USA) and 2 U of Taq DNA polymerase (AmpliTaq Gold^®^ DNA Polymerase, Applied Biosystems, Carlsbad, CA, USA).

**Table 1:** EGFR oligonucleotide sequences for PCR analysis

**Exon**	**Specific primers**	**PCR product**
**Exon 18**	F : 5^′^- GCT GAG GTG ACC CTT GTC TC -3^′^	225 bp
	R: 5^′^- CTC CCC ACC AGA CCA TGA -3^′^	
**Exon 19**	F: 5^′^- CAT GTG GCA CCA TCT CAC A -3^′^	230 bp
	R: 5^′^- CAG CTG CCA GAC ATG AGA A -3^′^	
**Exon 20**	F: 5^′^- CAT TCA TGC GTC TTC ACC TG -3^′^	377 bp
	R: 5^′^- CAT ATC CCC ATG GCA AAC TC -3^′^	
**Exon 21**	F: 5^′^- GCT CAG AGC CTG GCA TGA A -3^′^	348 bp
	R: 5^′^- CAT CCT CCC CTG CAT GTG T -3^′^	

F: *forward primer;* R: *reverse primer.*

The conditions for PCR were 95°C for 12 min, 40 cycles of 30 sec at 95°C, 45 sec at 65°C and 1 min at 72°C, followed by a cycle of 10 min at 72°C. After visualization of amplified products by gel electrophoresis on a 2% agarose, these were purified with USB^®^ ExoSAP-IT^®^ (Affymetrix, Inc., Cleveland, Ohio, USA). Subsequently the purified products were precipitated and labeled with GenomeLab™ DTCS Quick Start Kit (Beckman Coulter Inc, Fullerton, CA, USA) according to the manufacturer’s instructions and were sequenced using GenomeLab™ GeXP Genetic Analysis System (Beckman Coulter Inc, Fullerton, CA, USA). As described above, sequencing was performed in both directions, and the same PCR primers were used.

The sequences were analyzed using Genome Lab Genetic Analysis System v10.0.30 (Beckman Coulter Inc, Fullerton, CA, USA), and compared with the reference sequence ENSG00000146648.

#### Fluorescence *in situ* hybridization - *EGFR*

The ploidy status of *EGFR* in each tumor was assessed by fluorescence *in situ* hybridization (FISH) on previously constructed TMAs. Gene amplification was determined by using an *EGFR*/CEN-7 FISH Probe Mix (Y5500, Dako, Carpinteria, CA, USA) containing Texas Red-labeled DNA probe covering the full *EGFR* region and a mixture of fluorescein-labeled PNA probes targeted at the centromeric region of chromosome 7 (Chr7).

The FISH was performed using the Histology FISH Accessory Kit (Dako, Carpinteria, CA, USA), according to the manufacturer’s instructions.

Hybridization signals were visualized using fluorescence microscope equipped with a IAI monochrome progressive scan (IAI Company, Taiwan) and run by image analysis software Cytovision^®^ (Leica Microsystems, Wetzlar, Germany). 20 tumour nuclei/case were scored, and the tumor cells in which the signals of *EGFR* and CEP7 were increased equally were classified as polysomy 7 and those for which there was a double signal for *EGFR* or CEP7 were considered diploid. To assess gene amplification, we calculated the ratio of *EGFR* to CEP7 and evaluated in accordance with the criteria of FISH scoring system of Colorado Group [55]. Evaluation of the ploidy status of *EGFR* was performed by a single pathologist (EA).

### Statistical analysis

The association between the expression of TKR and ploidy of EGFR with different histological types of EST, as well the mutational status of TKR were assessed. All statistical analyses were performed based on contingency tables, with SPSS software v18.0 statistics (Chicago, Illinois).

## Results

### Classification of endometrial stromal tumors

From 65 EST cases, 80% (52/65) were diagnosed as ESS, and 20% (13/65) were UES. As expected, the majority of ESS presented expression of CD10, 51.9% (27/52), which was lower in UES cases (23.1%; 3/13).

### Analysis of expression of tyrosine kinase receptors

**Figure 1:**
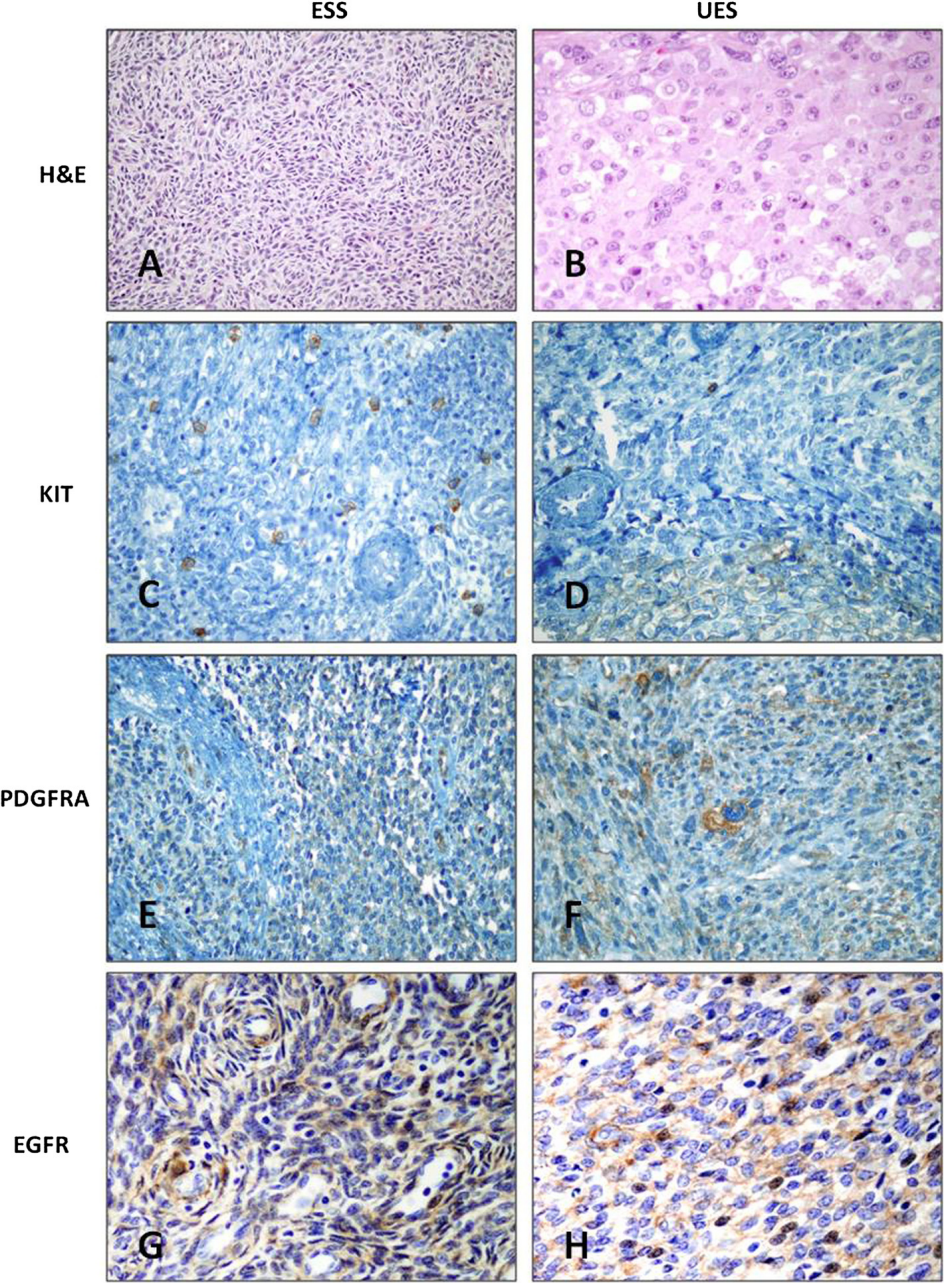
**Immunohistochemical expression of tyrosine kinase receptors in endometrial stromal tumors.** Endometrial stromal sarcoma, low grade (**A**) and undifferentiated endometrial sarcoma (**B**). Negative expression of KIT in ESS (**C**) and very focal expression in UES (**D**). Weak and/or focal PDGFRA and EGFR expression in ESS (**E, G**) and UES (**F,H**).

The expression of KIT, PDGFRA and EGFR was evaluated in all cases of EST, and an example of the pattern of expression of these markers is shown in Figure 1.

KIT stromal expression was found in only 2 out of 65 cases, and it was weak and/or focal. In contrast, PDGFRA showed positive stromal expression in 35.4% of cases (23/65), mostly weak and/or focal; its distribution was similar in both histological subtypes. Interestingly, expression was not intense and/or diffuse in any case. For EGFR, a minority of cases were classified as positive, representing 10.8% (7/65).

### Mutational status of tyrosine kinase receptors

The molecular study was performed in 62 cases. Sequencing of exons 9, 11, 13 and 17 of *KIT* only revealed the presence of a silent base substitution (ATC>ATT) in exon 17 at codon 798 (I798I) in two cases (ESS). The *PDGFRA* analysis showed the presence of several silent mutations, a base substitution (CCA>CCG) in exon 12 at codon 567 (P567P) in all cases, 32.3% (21/65) showed a silent base substitution (GTC>GTT) in exon 18 at codon 824 (V824V) and 1 case had a double silent base substitution at codons 824 and 838 (GTC>GTT and GGC>GGT; V824V and G838G respectively) both in exon 18. There were no somatic mutations in the genes *KIT* and *PDGFRA*.

*EGFR* mutational analysis was performed only in cases with expression of EGFR, and in which DNA was available for molecular studies. Two cases without EGFR expression were included as control. There were no mutations in *EGFR* exons 18, 19, 20 and 21. In 6 of 7 cases silent base substitution were identified at codon 787 (CAG>CAA; Q787Q), and in one of these cases were identified another silent base substitution at codon 790 (ACG>ACA; T790T) both in exon 20.

### *EGFR* amplification by FISH

*EGFR* gene amplification analysis was performed in all cases included in the study (65 cases), except in one which was considered not evaluable. No amplification of *EGFR* gene was observed. Numerous cases (58/64, 90.6%) were diploid (1–2 copies), and few cases (6/64, 9.4%) were polyploid (3–4 copies), and the distribution was similar in both histological types.

## Discussion

Systemic therapy in EST has a marginal efficacy. Low grade ESS can achieve control with hormonal treatments (progestins, aromatase inhibitors) and high grade undifferentiated uterine sarcomas are included in clinical trials together with leiomyosarcomas. Tanner *et al.* reported efficacy of docetaxel and gemcitabine or adriamycin for advanced cases of high grade endometrial tumors [56]. Indeed, the indication of systemic treatment of EST is controversial. Hormonal treatment can depend on hormonal receptor status and is associated to light side-effects and occasionally indicated. Responses were reported in a few ESS cases treated with progestin or aromatase inhibitors [57-64], but prospective larger trials are needed. Chemotherapy showed no apparent benefit in ESS [34,65-67]. In the case of UES some objective and partial responses were observed [68-70]. In a retrospective study including only 21 patients was described a response rate of 62% with gemcitabine/docetaxel and doxorubicin-based regimens in first-line, whereas in second- or additional chemotherapy for progressive disease, the response rate was around 19% [56]. On the other hand, radiotherapy seems to have a significant effect on local-regional control rate with a low impact on survival improvement [3,4,71-74]. Due to these facts several reports address the need to explore the role of TKR in these malignancies. The possible clinical efficacy of imatinib in the treatment of EST and the contribution of its TKR-target in disease progression are the key questions to solve [34].

Previous reports showed the presence at least one TKR in EST, but available data just refer a few case reports and small series, which makes it difficult to draw conclusions. Based on this, we performed an extensive evaluation of molecular and immunohistochemical expression of TKR KIT, PDGFRA and EGFR in a large series of ESS and UES.

KIT and PDGFRA cytoplasmic expression was observed in 3% and 35.4% respectively. In the literature, KIT expression has been reported to be quite variable (0-100%) (Table 2) and this might be due to differences in the choice of antibody clones, tissue pretreatment, sensitivity of immunohistochemical procedures, sample selection and use of whole tissue sections or TMA sections, which was also suggested in previous studies [75,76]. On the other hand, cytoplasmic PDGFRA expression in ESS, although mostly weak and/or focal, lies within the range reported in previous studies [34,37-39,77,78].

**Table 2:** TKR expression on endometrial stromal tumors

**Author**	**KIT (%)**		**PDGFRA (%)**		**EGFR (%)**	
	**ESS**	**UES**	**ESS**	**UES**	**ESS**	**UES**
Oliva, Young *et al.*[79]	0/8 (0)					
Hornick and Fletcher [80]	0/10 (0)					
Winter, Seidman *et al.*[81]	0/1 (0)					
Wang, Felix *et al*. [26]	3/11 (27)	2/3 (67)				
Rushing, Shajahan *et al*. [27]	2/2 (100)					
Klein and Kurman [28]	1/10 (10)	0/2 (0)				
Leath, Straughn *et al*. [29]	3/3 (100)					
Caudell, Deavers *et al*. [35]	0/8 (0)	1/4 (25)				
Moinfar, Gogg-Kamerer *et al*. [40]					14/20 (70)	3/3 (100)
Salvatierra, Tarrats *et al*. [36]		1/1 (100)				
Nakayama, Mitsuhashi *et al*. [76]	0/5 (0)					
Liegl, Gully *et al*. [38]	0/37 (0)		22/37 (59)			
Adams, Hickson *et al*. [39]	0/8 (0)		7/8 (88)			
Mitsuhashi, Nakayama *et al.*[42]		0/1 (0)				1/1(100)
Zafrakas, Theodoridis *et al*. [31]	2/2 (100)	1/2 (50)				
Martin, Ramesh *et al*. [32]	1/1 (100)					
Trojan, Montemurro *et al*. [37]	0/1 (0)		1/1 (100)			
Koivisto-Korander, Butzow *et al*. [33]	2/9 (22)					
Cheng, Yang *et al*. [34]	1/12 (8)		4/12 (33)			
Cossu-Rocca, Contini *et al*. [77]	0/23 (0)	1/5 (20)	15/23 (65)	4/5 (80)	10/23 (43)	4/5 (80)
Park, Kim *et al*. [78]	32/39 (82.1)		28/39 (71.8)		0/39 (0)	
Present series (2013)	1/52 (2)	1/13 (7.7)	18/52 (34.6)	5/13 (38.5)	6/52 (11.5)	1/13 (7.7)

In our series, mutational analysis of *hot spots* of *KIT* and *PDGFRA* genes did not reveal any somatic mutation, which is consistent with previous studies [36-39,41,42,77]. Constitutive activation via autocrine/paracrine stimulation of the receptor by its ligand was observed in several human cancers and is related with their tumorigenesis [82,83]. Probably, expression of related ligands of KIT and PDGFRA, which were not evaluated in this series, might be the reason for expression of both receptors in these tumors, although more studies are needed. Second, the work of Kang *et al.* demonstrated that relative KIT expression ratio was fivefold higher in cases with *KIT* mutation, than in GISTs lacking *KIT* mutation and the mutation status of *KIT* and *PDGFRA* was directly related to the different expression levels of activated KIT and PDGFRA [84]. Furthermore, with exception of GISTs, the presence of overexpression of KIT and PDGFRA in human cancers was not correlated with presence of activating mutations [85]. These facts support our results, once that KIT and PDGFRA expression observed was mostly weak and/or focal and lacks activating mutations on its related genes. Furthermore, the consistent expression of KIT and PDGFR receptors and their ligands was seen in a phase II trial in women with ovarian cancer, together with the absence of mutations in these genes and with lack of response to imatinib [86]. In other two phase II clinical trials in lung cancer [87] and uterine carcinosarcomas [88], the high expression of KIT did not correlate with response to imatinib. These clinical findings support the hypothesis that overexpression of these TKR does not confer sensitivity to imatinib, which was previously suggested for other uterine sarcomas [89].

However, the expression of at least one of imatinib-target (KIT, PDGFRA and PDGFRB) without evidence of activating mutations was reported in EST patients who responded to imatinib treatment [36,37,43]. Tumor shrinkage was the main indicator of objective response accompanied by stable disease in two cases [36,37]. One explanation for these results may be due to the autocrine/paracrine signaling of PDGFRB. In dermatofibrosarcoma protuberans (DFSP), *t*(17;22)(q22;q13) results in a *COL1A1-PDGFB* gene fusion. This cytogenetic abnormality is essential for pathogenesis of the disease and responsible by constitutive activation of PDGFRB. Imatinib inhibits the activity of the dysregulated PDGFRB [90], decreases enzymatic activity in DFSP cells and inhibits their ability to divide and grow [91] and induces apoptosis in tumor cells [92], which may have effects on decreasing tumor size [93]. In fact, an effect of imatinib on tumor shrinkage was observed in patients with DFSP [94,95], and therefore imatinib was approved for unresectable, recurrent, and/or metastatic DFSP in adults. However, in ESS and UES cases reported as responsive to imatinib, PDGFRB was only determined in one case reported by Trojan *et al.*[37], which makes difficult to draw any conclusions.

Concerning EGFR expression, our study reveals lower cytoplasmic expression of EGFR in EST cases (around 10.8%), in contrast with the results obtained by other authors [40,77] (Table 2). This contradiction may be a consequence of the same variables indicated for the immunoexpression of KIT. Since i) a correlation between an increased *EGFR* copy number and gefitinib was proposed in non-small-cell lung cancer (NSCLC) [96] and ii) immunohistochemistry is not a reliable approach for this determination, we decided to evaluate *EGFR* amplification status in our series by FISH. The *EGFR*/Chr7 ratio was below 2, which is indicative of non-amplification, and only a small percentage of the cases presented polysomy. Previous evaluation of *EGFR* amplification was only performed in a UES case which presented a temporary response to imatinib. This case presented a low-level amplification (mean ratio 2.9) according to the evaluation criteria of the authors [42]. Although no consensus has been reached on how to assess the presence and extent of *EGFR* status dysregulation in solid tumors by FISH analysis [97], it seems likely that gene amplification found in NSCLC and glioblastoma is an uncommon event in EST. *EGFR* mutational status also did not show any somatic mutation in exons 18–21. In fact, all these data confirm that it is unlikely that EGFR activation could play a role in tumorigenesis of EST.

## Conclusions

In summary, we performed an extensive molecular and expression analysis of KIT, PDGFRA and EGFR in the largest series of EST studied so far (Table 2). Our findings reveal a lack of significant expression, amplification and activating mutations on these receptors and related genes, which suggests that it is unlikely that EST can benefit from therapies such as imatinib or EGFR inhibitors in advanced disease. However, the role of imatinib in the management of unresectable tumors needs to be clarified through evaluation of expression of constitutive activation of PDGFRB by its ligands, or other genetic alterations in ESS and UES. The recent discovery of a chromosomal rearrangement *t*(10;17)(q22;p13) that results in a 14-3-3 fusion protein with oncogenic properties in a subgroup of UES [22], and the nuclear expression of β-catenin, a member of Wnt and E-cadherin signaling pathways in EST need to be better explored, since they can represent an entry point for targeted therapeutic strategies.

## Competing interests

Support for this study was obtained from Novartis.

## Authors’ contributions

RS carried out the molecular genetic studies, performed the sequence alignment and data analyses and drafted the manuscript. TH and SF carried out the TMA construction and immunohistochemistry assays. TH carried out fluorescent hybridization assay and participated in the sequence alignment. FT, AV, MCG, AA, NH, JS, JO, LG and RR provided the samples for the study. CB and EA conceived of the study, and participated in its design and coordination and helped to draft the manuscript. All authors read and approved the final manuscript.
